# Transcranial Direct Current Stimulation in Anorexia Nervosa: A Randomized Sham-Controlled Trial

**DOI:** 10.3390/jcm15176831

**Published:** 2026-09-03

**Authors:** Zuzanna Rząd, Joanna Rog, Natalia Kajka, Klaudia Kister, Paweł Szewczyk, Anna Rymuszka, Anna Sierosławska, Michalina Hordejuk, Hanna Karakuła-Juchnowicz

**Affiliations:** 1 Doctoral School, Medical University of Lublin, 7 Chodźki Street, 20-093 Lublin, Poland; klaudia2178@gmail.com (K.K.); pawel.szewczyk33@gmail.com (P.S.); 2 Department of Basic Medical Sciences, Faculty of Medicine, The John Paul II Catholic University of Lublin, 1 H Konstantynów Street, 20-708 Lublin, Poland; 3 1st Department of Psychiatry, Psychotherapy and Early Intervention, Medical University of Lublin, Gluska Street 1, 20-439 Lublin, Poland; natalia.kajka@umlub.pl (N.K.); michalina.hordejuk@umlub.edu.pl (M.H.); hanna.karakula-juchnowicz@umlub.edu.pl (H.K.-J.); 4 Department of Physiology and Toxicology, Faculty of Medicine, The John Paul II Catholic University of Lublin, 1 H Konstantynów Street, 20-708 Lublin, Poland; anna.rymuszka@kul.pl; 5 Department of Preclinical Sciences, Faculty of Medicine, The John Paul II Catholic University of Lublin, 1 H Konstantynów Street, 20-708 Lublin, Poland; anna.sieroslawska@kul.pl

**Keywords:** anorexia nervosa, transcranial direct current stimulation, neuromodulation, dorsolateral prefrontal cortex, eating attitudes test

## Abstract

**Background/Objectives**: Anorexia nervosa (AN) remains difficult to treat, and evidence for biological adjunctive interventions is limited. This study assessed the effectiveness of transcranial direct current stimulation (tDCS) as an adjunctive intervention in hospitalized patients with AN. **Methods**: In this double-blind, randomized, sham-controlled trial, 40 female inpatients with AN were allocated 1:1 to active tDCS or sham stimulation. Participants received 30 sessions over 3 weeks (2 mA, 25 min, twice daily on weekdays; anode F3, cathode F4), alongside standard inpatient treatment. Assessments were performed at baseline, post-intervention, and 2-week follow-up. Outcomes included eating disorder (ED) symptoms (EAT-26), depressive symptoms (BDI), perceived stress (PSS-10), self-esteem (SES), body mass index (BMI), and selected biological markers. **Results**: EAT-26 scores improved significantly from baseline to post-intervention in both groups; at the 2-week follow-up, scores remained significantly lower than baseline only in the active tDCS group. BMI and leptin increased significantly in both groups, while NT-3 decreased only in the active group. In multivariate analyses, higher leptin levels and greater perceived stress, adjusted for longer illness duration, were associated with greater improvement in ED symptoms; perceived stress predicted symptom improvement only in the active group. **Conclusions**: Active tDCS did not produce significantly greater short-term symptom reduction than sham in hospitalized patients with AN. However, findings observed at the 2-week follow-up and associations involving stress-related and metabolic measures require confirmation in studies with longer follow-up periods.

## 1. Introduction

Anorexia nervosa (AN) is a mental disorder found in all countries and socioeconomic groups worldwide [1]. According to the Global Burden of Disease 2021 estimates, the prevalence of AN remains highest in high-income Western regions such as North America, Western Europe, and Australasia. Countries such as Asia, Africa, and most Latin American countries report relatively lower incidence rates [2]. It is a complex and multidimensional eating disorder recognized in both the International Classification of Diseases (ICD-11) and the Diagnostic and Statistical Manual of Mental Disorders (DSM-5-TR) [3,4]. AN most often begins in adolescence, and epidemiological data indicate that it may affect 0.8 to 6.3% of women and 0.1 to 0.3% of men over the course of their lives [5,6]. The clinical presentation of the disease is often heterogeneous, with patients requiring hospitalization and multifaceted medical interventions [7]. The most commonly used treatment methods include nutritional rehabilitation, individual psychotherapy, family therapy, dietary counseling, and pharmacotherapy [7,8]. It should be emphasized, however, that there are currently no licensed and approved biological treatments for AN [9]. Furthermore, the effectiveness of AN treatment remains limited—only about one-third of adolescents experience temporary improvement [8], relapses occur in 9–52% of patients, the average duration of the disease is approximately 6 years, and 30–60% achieve long-term remission, while approximately 20% remain chronically ill [10].

There is a clear need to explore new therapeutic strategies that can complement standard treatment and increase the durability of clinical improvement in patients with AN. Interventions targeting mechanisms that are insufficiently addressed in current psychotherapeutic and pharmacological approaches may be particularly important. One non-invasive and safe method is transcranial direct current stimulation (tDCS), which has rarely been studied in individuals with AN [11,12,13]. This is supported by a recent meta-analysis by Ramanathan et al. [14], which presented only four studies, and preliminary results suggest potential improvements in nutritional status. Although researchers point to potential improvements in body mass index (BMI) and reductions in symptoms of AN, anxiety, and depression with tDCS, the methodological limitations of these studies (small sample size, lack of appropriate control groups) prevent drawing firm conclusions. In turn, a study by Strumila et al. [13] demonstrated the efficacy and safety of tDCS in the treatment of psychiatrically hospitalized patients with anorexia nervosa.

Although available research indicates preliminary efficacy and safety of tDCS in small clinical trials, data on the mechanisms by which this method modulates cognitive and emotional processes in patients with anorexia nervosa are still lacking [12]. In particular, it is unknown whether the effects of tDCS result from changes in cortical neuroplasticity (e.g., modulation of neurotrophins such as brain-derived neurotrophic factor (BDNF)), modulation of hypothalamic-pituitary–adrenal (HPA) axis reactivity, or direct effects on corticolimbic networks involved in cognitive control and reward regulation. The limited number of studies also precludes drawing clear conclusions regarding the dose-dependent effect and the number of stimulation sessions, as well as whether biological changes precede or follow clinical improvement. The use of a randomized, double-blind, sham-controlled trial is the gold standard for assessing the effectiveness of neuromodulatory interventions, allowing for control of placebo effects and other nonspecific factors, such as the impact of the hospitalization environment. Therefore, the aim of this study was to verify the clinical efficacy of tDCS and examine its potential role as a modulator of the processes underlying treatment response. Previous studies clearly indicate that a particularly important stimulation region is the dorsolateral prefrontal cortex (DLPFC), which plays a key role in the excessive cognitive control and rigid behavioral patterns observed in anorexia nervosa [11,13,15]. The DLPFC plays a key role in response inhibition, cognitive flexibility, and top-down regulation of reward-related behaviors—processes directly linked to core clinical symptoms of AN, including food avoidance and excessive dietary restraint [8]. Functional neuroimaging studies have shown greater DLPFC activation in AN patients compared with healthy controls during exposure to food-related stimuli and food choice tasks [16], consistent with broader evidence for hyperactivation in regions responsible for cognitive control in AN. tDCS is hypothesized to modulate cortical excitability and influence neural networks involved in cognitive control, emotion regulation, and reward processing [17]. Furthermore, a meta-analysis by Wang et al. demonstrated that tDCS influences neuroplasticity processes, including through the modulation of neurotrophins such as BDNF [18]. It is a key regulator of synaptic plasticity and neuronal survival; its serum levels are often decreased during the acute phase of AN and are suggested to normalize with nutrition and clinical recovery [19,20,21,22]. Therefore, in the present study, we hypothesized that active stimulation would be associated with a greater increase in BDNF compared to sham stimulation.

Neurotrophins structurally related to BDNF and involved in neuronal differentiation and survival, along with nerve-growth factor (NGF), were assessed in an exploratory manner in this study. Given the limited existing literature directly linking these markers to tDCS, they were included to characterize the broader neurotrophic response to stimulation and have additionally been linked to obesity and low-grade inflammation, which are significantly altered during nutritional rehabilitation in AN [23]. Leptin and visfatin reflect nutritional and metabolic status. In the present study, they were also assessed in an exploratory manner. Leptin is a well-known marker of weight regain in AN and is additionally associated with mood regulation during recovery [24,25,26]. However, visfatin was included in addition to leptin as a marker of adipose tissue activity during refeeding, given evidence that its concentration remains altered in AN and does not normalize after partial weight regain [27]. No tDCS-specific directional hypotheses were formulated a priori for these five markers. Associations with clinical and anthropometric variables are presented as exploratory results.

In turn, research by Mehrsafar et al. [28] shows that tDCS can influence stress-related systems, including the HPA axis. These findings suggest that tDCS may modulate mechanisms underlying treatment response rather than exerting a direct symptomatic effect. Therefore, it is postulated that active stimulation will be associated with greater cortisol reduction compared to sham stimulation.

The aim of this study was to evaluate the effectiveness of tDCS as an adjunctive therapeutic intervention in hospitalized patients with AN compared with sham stimulation and to examine its impact on emotional processes associated with treatment response in a randomized, double-blind clinical trial. tDCS was evaluated primarily as an adjunctive treatment for eating disorder symptoms, while depressive symptoms, perceived stress, and self-esteem were assessed as secondary, clinically related dimensions of psychological functioning.

## 2. Materials and Methods

### 2.1. Trial Design and Intervention

The present study reports the final results of a randomized, sham-controlled clinical trial whose protocol and preliminary findings have been published previously [8,12].

The non-prespecified interim analysis was exploratory and was not used for formal efficacy stopping or modification of the trial design. Access to unblinded data was restricted to the data analyst, while personnel involved in recruitment, intervention delivery, and outcome assessment remained blinded. Recruitment continued to the predefined sample size without changes to the protocol, and the final conclusions were based on the complete trial dataset.

The study was designed in accordance with the Standard Protocol Items: Recommendations for Interventional Trials (SPIRIT) 2025 guidelines for interventional trials and is reported in line with the CONsolidated Standards Of Reporting Trials (CONSORT) 2025 statement [29,30] (CONSORT 2025 checklist is presented in Supplementary Materials). Ethical approval was obtained from the Bioethics Committee of the Medical University of Lublin (approval number: KE-0254/24/01/2022). The trial was registered at ClinicalTrials.gov (identifier: NCT05814458) and conducted in line with the principles of the Declaration of Helsinki and Good Clinical Practice. After trial initiation, the eligibility age range was expanded. and additional assessment measures were introduced to enhance data collection. These changes were implemented during the recruitment phase and did not affect the primary outcome or core intervention procedures. Patients and the public were not involved in the design, conduct, reporting, or dissemination of this trial.

Participants received either active or sham tDCS twice daily on weekdays over a three-week period, yielding a total of 30 stimulation sessions. Each session lasted 25 min, with a two-hour interval between consecutive stimulations. In the active stimulation condition, a constant current of 2 mA was delivered via saline-soaked sponge electrodes positioned over the dorsolateral prefrontal cortex. Electrode placement followed the International 10–20 system, with the anode located at F3 and the cathode at F4. Current intensity was gradually increased over 30 s at the beginning of each session and decreased over 30 s at the end. Sham stimulation was administered using the same electrode montage and device settings. To maintain participant blinding, the device briefly ramped up to the target intensity before reducing the current to a minimal test level (25 µA), which was maintained throughout the session to monitor electrode contact. At the conclusion of each session, the ramp-up and ramp-down procedure was repeated, with both phases lasting 30 s. The intervention protocol, stimulation intensity, electrode size, current density, and electrode montage was identical for all participants, and no modifications to stimulation parameters were permitted during the trial. All sessions were conducted under direct supervision, ensuring full adherence to the intervention protocol. All stimulation sessions were conducted using the Soterix Medical Inc. (Woodbridge, NJ, USA) 1 × 1 tDCS mini-CT LTE device (model 1601-LTE). A follow-up assessment was performed two weeks after completion of the stimulation protocol.

### 2.2. Participants

Participants were recruited consecutively from eligible female inpatients hospitalized for AN at the 1st Department of Psychiatry, Psychotherapy, and Early Intervention of Medical University in Lublin, Poland. Written informed consent was obtained from all participants using a single consent form. For participants under 18 years of age, the form included separate signature fields for both the minor and their parent or legal guardian, both of which were required. Participants aged 18 years or older signed independently, without requirement for parental or guardian consent.

Eligibility was limited to females aged between 10 and 30 years who met the DSM-5 diagnostic criteria for AN and presented with a BMI of 17.5 kg/m^2^ or lower.

With regard to concomitant treatment, participants were not permitted to receive psychotropic medications other than antidepressants, including SSRIs, SNRIs, and TCAs. Participants receiving antidepressants were eligible if both the medication and dose had remained unchanged for at least one month before screening. No changes in psychopharmacological treatment were allowed during the intervention or follow-up period. The antidepressants and daily doses used in each study group are presented in Table 1. Treatment duration beyond the required one-month stabilization period was not systematically recorded.

Exclusion criteria included a history of neurological or organic disorders (e.g., epilepsy), any contraindications to transcranial direct current stimulation, such as the presence of pacemakers or metal implants in the cranial area, and the occurrence of psychiatric comorbidities other than mood disorders considered secondary to AN. Pregnancy, plans to become pregnant during the study period, and any modifications in psychopharmacological treatment over the course of the trial also constituted grounds for exclusion.

### 2.3. Randomization, Allocation Concealment, and Blinding

At study entry, eligible participants were randomly assigned to one of two parallel groups: an active tDCS group (AG; *n* = 20) or a sham stimulation group (SG; *n* = 20). Randomization was performed independently of clinical and demographic characteristics, using a computerized permuted block randomization procedure (Research Randomizer, Version 4.0; Urbaniak GC, Plous S., 2013) with a 1:1 allocation ratio. Group assignment was conducted by a researcher not involved in either stimulation delivery or outcome assessment, ensuring appropriate allocation concealment.

The study was developed by the academic research team. Study oversight was ensured by an independent external data and safety monitor (J.R.), who was responsible for periodic safety reviews conducted during the course of the trial. To preserve blinding, J.R. was not involved in any patient-related procedures, stimulation sessions, or data acquisition and had access only to a limited set of unblinded data required for safety monitoring. All investigators remained blinded to group allocation throughout the study.

In addition, device codes for active and sham stimulation were programmed by a separate researcher (M.H.), who did not participate in patient recruitment, clinical assessments, or data analysis. Allocation was concealed using pre-generated randomization codes accessible only to the independent researcher. This procedure ensured double blinding at the level of both the participants and the principal investigator (Z.R.). The researchers responsible for administering the stimulation were Z.R., K.K., and P.S.

### 2.4. Recruitment

**Figure 1:**
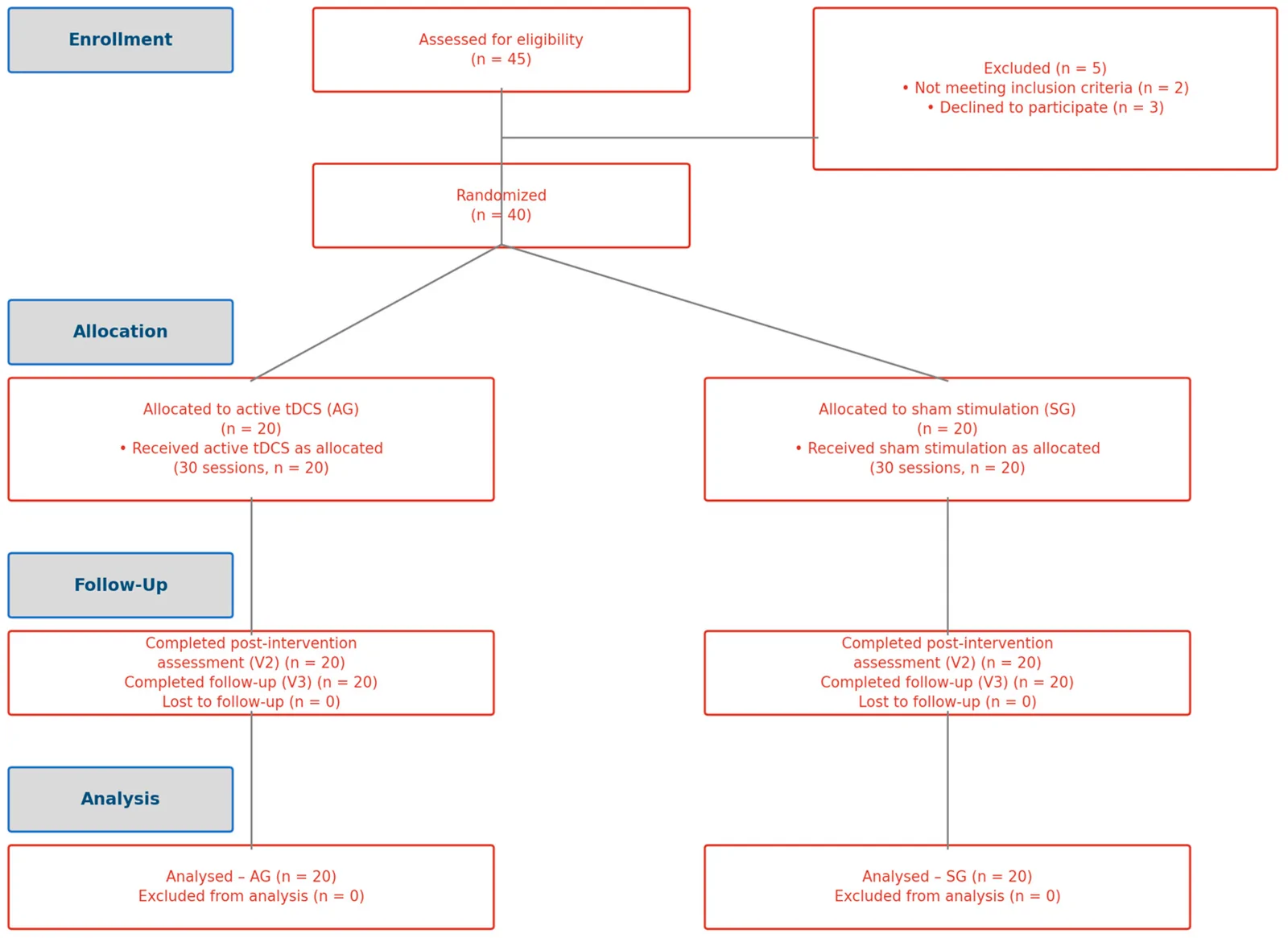
Consort flow diagram.

The trial was conducted from October 2023 to September 2025. A total of 45 individuals were screened: 2 did not meet inclusion criteria and 3 declined to participate. The remaining 40 patients were randomly assigned (20 each) to the AG or SG. Recruitment of participants for the study was stopped after the predetermined sample size was reached. Consequently, the effective sample size of the study was 40 (20 in each group). All randomized participants completed the intervention and received the intervention as planned. The flow of participants through the study is presented in accordance with the CONSORT statement. Details are provided in Figure 1.

The sample size was determined a priori, as described in the published study protocol [8]. The calculation was based on previous trial data indicating improvement in EAT-26 scores and assumed a baseline score of 72.7 points and an anticipated 20-point improvement. A target sample of 40 evaluable participants (20 per group) was estimated with a statistical power of 80%. To account for an anticipated dropout rate of 20%, the protocol allowed for the recruitment of up to 50 participants. As all 40 randomized participants completed the intervention, the predefined target number of evaluable participants was achieved.

### 2.5. Study Timeline and Outcome Measures

The study followed a predefined schedule comprising four study visits. The screening visit (V0) took place 1–2 days prior to baseline and was used to verify eligibility criteria and enroll participants into the study. The baseline visit (V1), conducted one day before the initiation of stimulation, included randomization and completion of baseline assessments. The post-intervention visit (V2) was scheduled at the end of the stimulation period, up to three days after completion of the three-week intervention (within 3 days after completion of the intervention). A follow-up visit (V3) was performed approximately 2 weeks after the post-intervention visit (V2) (±3 days) to complete all remaining study procedures.

A detailed description of all assessment tools applied in the trial has been published previously in the study protocol [8]. For the purposes of the present analysis, which reports the final study outcomes, a selected set of measures was included.

Sociodemographic and clinical background data were collected using a self-developed questionnaire covering personal information, medical history, menstrual status, and current pharmacotherapy. The prespecified primary outcome was change in the EAT-26 total score. BMI and the Beck Depression Inventory (BDI), Perceived Stress Scale (PSS-10), and Rosenberg Self-Esteem Scale (SES) scores were treated as secondary clinical outcomes, whereas the biological markers were analyzed as exploratory outcomes.

Eating disorder symptoms were assessed using Part A of the EAT-26, a well-established standardized self-report screening measure [31]. The EAT-26 was selected because its emphasis on eating-related attitudes and concerns was considered suitable for assessing patients receiving treatment in a structured inpatient setting. The prespecified primary outcome was the change in the EAT-26 total score from baseline (V1) to the end of the three-week stimulation period (V2), as defined in the published study protocol [8]. The V3 assessment was a follow-up evaluation intended to examine the maintenance of changes after completion of the intervention. In addition to the total score, three subscales were analyzed: Dieting, Bulimia and Food Preoccupation, and Oral Control.

Psychological functioning was evaluated using validated self-report questionnaires, including the BDI [32] to assess depressive symptom severity, the Perceived Stress Scale (PSS-10) to measure subjectively experienced stress [33], and the SES, which captures global self-esteem as a relatively stable personal disposition [34].

Anthropometric assessment included measurements of body weight and calculation of BMI. Safety and tolerability of the intervention were assessed repeatedly during the intervention period using the tDCS Side Effects Questionnaire [35].

In addition, biological parameters were assessed based on venous blood samples collected at two time points: before the initiation of stimulation (V1) and immediately after completion of the stimulation protocol (V2). Laboratory analyses included selected neuroendocrine and metabolic markers: BDNF, neurotrophic factors neurotrophin-3 (NT-3) and neurotrophin-4 (NT-4), leptin, visfatin, NGF, and cortisol. All biological markers were quantified in venous blood samples using commercially available enzyme-linked immunosorbent assay (ELISA) kits according to the manufacturers’ instructions.

**Figure 2:**
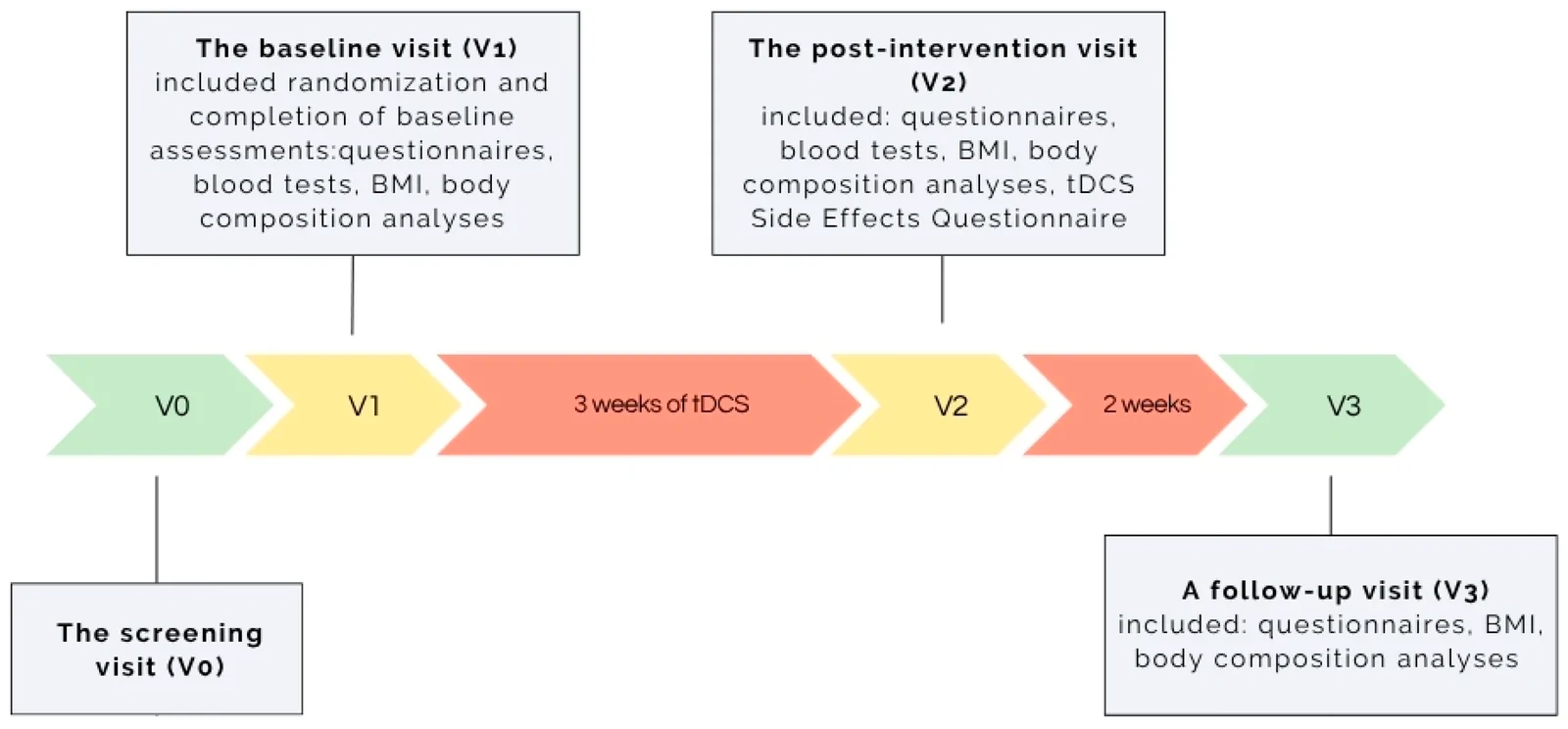
Timeline of the study.

Self-report measures were completed by participants, while anthropometric and laboratory outcomes were assessed by trained clinical staff blinded to group allocation. The timeline of the study is presented in Figure 2.

### 2.6. Statistical Analysis

Statistical analyses were conducted using Statistica version 13 (StatSoft, Inc., TIBCO). Statistical significance was set at *p* < 0.05 for all analyses. The distribution of quantitative variables was assessed using the Shapiro–Wilk test. Descriptive statistics were calculated for sociodemographic variables, with mean (X) ± standard deviation (SD) for normally distributed variables or median (Me) and interquartile range (IQR) for non-normally distributed variables and percentages for categorical variables.

The relationship between biological variables, symptom severity, and anthropometric measurements at V1 was assessed using Spearman’s rank correlation.

To examine differences in the studied variables across three time points, a mixed-design ANOVA with repeated measures was performed. For comparisons between two time points (V0-V1), Student’s *t*-test was used for normally distributed variables, and the Wilcoxon signed-rank test was used for non-normally distributed variables. Between-group differences at each time point (V1, V2, V3) were assessed using Student’s *t*-test for normally distributed variables and the Mann–Whitney U test for non-normally distributed variables.

To investigate the effect of selected predictors on the primary study outcome, change in EAT-26 scores (ΔEAT-26, calculated as V2 − V1) was used as the dependent variable in a multiple stepwise regression analysis. A stepwise procedure was applied for final variable selection. Baseline variables included in the initial model were: BMI, biological variables (neurotrophins and cortisol levels in blood), time since last menstruation, duration of illness, BDI, PSS-10, and SES scale total points.

## 3. Results

### 3.1. Sociodemographic and Clinical Characteristics of the Participants

The characteristics of the enrolled individuals are presented in Table 1. The age of participants ranged from 12 to 27 years at study entry (median: 14), with no significant differences between study groups (*p* > 0.05). The mean BMI at study entry and during hospitalization and the lowest body mass during illness did not differ between groups (*p* > 0.05). Most individuals presented the restrictive subtype of AN (*n* = 32, 80%), with no differences between study groups (*p* > 0.05).

**Table 1:** Baseline characteristics of the participants.

Variables	AG	SG	*p*-Value
Age	15.15 ± 2.6	15 ± 3.16	0.826
BMI (kg/m^2^)	15.06 ± 1.12	15.13 ± 1.314	0.847
Body weight (kg)	39.32 ± 3.19	39.83 ± 4.701	0.686
Duration of hospitalization (days)	94.16 ± 23.43	96.23 ± 33.05	0.821
Duration of illness (months)	19.65 ± 26.09	26.33 ± 39.15	0.327
Lowest body weight (kg)	35.5 ± 4.11	35.33 ± 4.04	0.896
Highest body weight (kg)	52.05 ± 7.21	54.95 ± 13.16	0.969
Time since last menstruation (months)	8.89 ± 8.4	9.13 ± 7.01	0.514

Data presented as mean and standard deviation; AG—active group, SG—sham group.

Thirteen participants in the AG (65%) and 18 participants in the SG (90%) received at least one antidepressant. SSRIs were the most commonly used antidepressants, with sertraline being the most frequent. The distribution of individual medications and daily doses by study group is presented in Table 2. As some participants received more than one antidepressant, medication-specific counts are not mutually exclusive, and their sum may exceed the number of participants in a given study group. Antidepressant treatment remained unchanged throughout the study.

Chronic comorbid conditions other than AN were present in six participants in the AG and four participants in the SG. The median total score on the EAT-26 scale was 39 (range: 1–59) and 19 (range: 0–67) points in the AG and SG, respectively, with no significant differences between groups (*p* > 0.05). No differences were observed in other symptoms based on the PSS-10 scale (24 in the AG and 23 in the SG), the BDI scale (19 in the AG and 20 in the SG), or the SES (23 in the AG and 24 in the SG) (*p* > 0.05).

### 3.2. Relationships Between Biological, Clinical, and Anthropometric Variables at Study Entry

We found several associations between biological variables and the clinical picture as well as body composition variables at V1. In particular, NGF was positively associated with body mass (expressed in kg) (R = 0.33, *p* < 0.05) and fat mass (R = 0.32, *p* < 0.05) and negatively associated with fat-free mass (expressed in kg) (R = −0.34, *p* < 0.05). NT-3 and visfatin were inversely associated with the length of amenorrhea (R = −0.40, *p* < 0.05 and R = −0.38, *p* < 0.05, respectively). BDNF was positively associated with the percentage of extracellular water (R = 0.51, *p* < 0.05) and inversely associated with the percentage of intracellular water (R = −0.51, *p* < 0.05).

### 3.3. Changes in the Primary Eating Disorder Outcome and Secondary Psychological Outcomes

At baseline (V1), no significant between-group differences were observed in any of the examined clinical outcomes. Table 3 presents the results of the repeated-measures ANOVA.

For the prespecified primary outcome, no significant group × time interaction was observed for the EAT-26 total score (*p* = 0.450). EAT-26 scores decreased significantly from V1 to V2 in both groups (AG: *p* = 0.023; SG: *p* = 0.006); however, a significant difference between V1 and V3 was observed only in the AG (*p* < 0.001).

Regarding the secondary psychological outcomes, no significant group × time interactions were found for depressive symptoms, self-esteem, or perceived stress (BDI: *p* = 0.189; SES: *p* = 0.156; PSS-10: *p* = 0.787). BDI scores decreased significantly in the SG between V1 and V2 (*p* = 0.005) and between V1 and V3 (*p* = 0.002). No other significant within-group changes were observed for the secondary psychological outcomes.

### 3.4. Changes in Exploratory Biological Outcomes and BMI Following tDCS

In both groups, significant differences in leptin levels were observed between V1 and V2 (*p* < 0.001 for both groups). Leptin levels increased from 3.543 (0.183–13.815) to 9.782 (0.432–54.612) in the AG and from 3.090 (0.183–53.733) to 14.784 (0.183–180.897) in the SG. In the AG, a significant change in NT-3 levels was observed between V1 and V2 (*p* = 0.012), with levels decreasing from 4.84 (3.28–34.3) to 4.41 (3.64–32). Differences in biological markers and BMI following tDCS are presented in Table 4 and Table 5 as medians with minimum and maximum values and interquartile range.

### 3.5. Determinants of Treatment Response

Treatment response was defined as a change of 30% or more in the EAT-26 score between V1 and V2. Overall, no differences were observed between responders and non-responders in biological markers, symptom severity, or anthropometric measurements (*p* > 0.05). However, in the SG group, we observed differences in baseline NT-4 levels between responders and non-responders (*p* = 0.017). Moreover, in this subgroup (SG non-responders), associations were found between biological markers measured at V0 and AN symptoms as well as body weight. Specifically, the total EAT-26 score was positively correlated with NGF (R = 0.501; *p* < 0.05) and visfatin (R = 0.482; *p* < 0.05). Body weight was also positively correlated with NGF (R = 0.623; *p* < 0.05) and NT-3 (R = 0.501; *p* < 0.05).

### 3.6. Factors Associated with the Primary Study Outcome

Multivariate models included the following variables: BMI, biological variables (blood levels of neurotrophins and cortisol), time since last menstruation, duration of illness, and total scores on the BDI, PSS-10, and SES scales. The final model indicated that leptin levels and perceived stress were significantly associated with changes in AN symptoms. Higher leptin levels and greater perceived stress, adjusted for longer illness duration, were associated with greater improvement in AN symptoms, explaining approximately 29.8% of the variance (F(3,28) = 5.389, *p* < 0.005). In subgroup analyses, only in the AG group was perceived stress identified as a significant predictor of improvement in AN symptoms.

### 3.7. Safety

In our sample of 40 girls (20 active, 20 sham), six participants (30%) in the active group and three participants (15%) in the sham group reported mild adverse events, most commonly transient headaches (active: four; 20%, sham: two; 10%) and dizziness (active: three; 15%, sham: 1; 5%), with one participant in the active group reporting both symptoms. All events were short-lived, resolved spontaneously without intervention, and no serious or long-term adverse effects were observed. No participant discontinued the study due to adverse events.

## 4. Discussion

The present randomized, double-blind, sham-controlled trial evaluated whether active tDCS provided additional clinical benefit when added to intensive multidisciplinary inpatient care, rather than whether tDCS was effective as a stand-alone treatment for AN. Although clinical improvement was observed during hospitalization, active stimulation was not associated with a significantly greater reduction in eating disorder symptoms than sham stimulation. Both groups demonstrated improvement in EAT-26 scores and changes in selected biological parameters. Therefore, under the conditions of the present study, active tDCS did not demonstrate an incremental short-term benefit beyond the effects of intensive inpatient treatment.

The observed improvement in both groups is consistent with the multifactorial nature of treatment response in AN. In the inpatient setting, clinical change results from the combined effects of nutritional rehabilitation, structured daily routine, psychotherapy, and environmental stabilization [36]. Partial improvement of nutritional status may influence mood, cognition, and emotional regulation, contributing to gradual symptom reduction independent of additional interventions. Therefore, the potential effects of neuromodulation should be interpreted within the context of an intensive multimodal treatment program where nonspecific and treatment-related factors may substantially contribute to clinical improvement.

Although no significant group × time interaction was observed, EAT-26 scores at the 2-week follow-up were significantly lower than baseline only in the AG. This within-group finding does not establish a treatment-specific maintenance effect, particularly given the short follow-up period and the absence of a significant group × time interaction. It should therefore be regarded as exploratory and hypothesis-generating. Consistent with our findings, another study reported that reductions in certain psychopathological measures and improvements in nutritional status were maintained at the 1-month follow-up following treatment for AN [11]. In obsessive-compulsive disorder, a single study demonstrated a counterintuitive effect: tDCS transiently reduced symptoms, but these gains were not maintained at follow-up, emphasizing the importance of strategies for sustaining treatment efficacy.

The analyses performed in the present study indicate that improvement in AN during hospitalization is a multidetermined process in which metabolic factors and subjectively experienced stress play an important role. In the multivariate model, higher leptin levels and greater perceived stress were associated with greater reduction in eating disorder symptoms. In AN, recovery is associated with increased leptin levels, which have been linked to reductions in depressive symptoms, suggesting a role for leptin in mood regulation [24,25]. Paradoxically, some studies have shown that higher leptin levels during early recovery are associated with increased depression, anxiety, and stress. Therefore, the relationship between leptin and psychological symptoms may vary depending on the severity of the disorder [26].

The association between perceived stress and AN symptoms changes suggest that stress-related mechanisms may influence treatment responsiveness. The majority of available evidence indicates that psychological stress is associated with reduced adherence to treatment guidelines and prescribed regimens [37,38]. However, awareness of disease severity has been proposed as a potential motivator of treatment adherence [39]. Nevertheless, this association has not been demonstrated in eating-behavior-related problems, particularly among underage patients. These findings suggest that tDCS may modulate mechanisms underlying treatment response rather than exerting a direct symptomatic effect. This interpretation is consistent with the hypothesis that prefrontal stimulation primarily affects cognitive control and emotional regulation rather than core eating disorder behaviors. An additional exploratory finding concerns the associations between neurotrophic factors and clinical as well as anthropometric variables. We observed positive correlations between EAT-26 scores and NGF and visfatin levels, as well as between body weight and NGF and NT-3 concentrations. Although these results should be interpreted with caution due to the relatively small sample size, assessment of peripheral biomarkers, and the exploratory nature of the analyses, they may suggest that neurotrophic and metabolic factors are related to the clinical severity of AN. Numerous studies have demonstrated reduced BDNF concentrations in individuals with an acute phase of AN [19,20,21,22]. However, our study did not observe significant changes in this neurotrophin during recovery and tDCS treatment. One study suggested that partial recovery may be insufficient to increase BDNF levels, and the authors proposed that alterations in serum BDNF in patients with AN are not solely attributable to changes in body weight [20]. On the other hand, a longer recovery period may be required to observe changes in peripheral BDNF levels. To the best of our knowledge, no study has specifically examined NGF in AN. Vigorous physical activity has been shown to exhibit a direct, positive longitudinal relationship with NGF in children [40]. Additionally, percent of body fat was positively associated with NGF concentrations in children and was found to be approximately 1.4-fold higher in overweight or obese women [41]. These findings may suggest a potential association between NGF and inflammatory processes, as reflected by links with physical activity and body fat. NT-3 has been studied in contexts such as brain-related disease; however, its specific role in AN remains unexplored. In psychiatric disorders, elevated NT-3 levels have been observed in patients with schizophrenia with coexisting depressive symptoms and during depressive and manic episodes in bipolar disorder [42,43]. Antidepressants and mood stabilizers may contribute to the normalization of NT-3 levels [44]. Additionally, acute resistance training and combined exercise have been shown to increase NT-3 levels in the population of overweight individuals [45]. Therefore, hypothetically, NT-3 may be involved in neurobiological adaptations related to metabolic status, physical activity, and affective dysregulation in AN.

Therefore, the present study provides preliminary data indicating that these parameters may be relevant for understanding individual differences in treatment response and the neurobiological mechanisms underlying recovery.

Current evidence regarding the efficacy of tDCS in AN remains limited and inconsistent. According to our knowledge, three studies have been published to date evaluating the efficacy of tDCS in AN. Two of them reported significant reductions in both depressive symptoms and eating disorder severity, whereas one study did not confirm these effects; notably, this was the only sham-controlled trial conducted prior to our study [1,13,15]. In addition, one study compared active tDCS with family psychotherapy and reported greater improvement in the tDCS group [11]. Previous studies did not include biological outcome measures other than BMI and typically used 10–20 stimulation sessions. Against this background, the present study extends the limited sham-controlled evidence by evaluating a longer, 30-session protocol and showing that it did not provide a measurable additional short-term benefit over intensive multidisciplinary inpatient care.

The results of our interim analysis were generally consistent with earlier findings, showing improvement in eating disorder symptoms in the active stimulation group, while no clear changes were observed in the sham group, and BMI changes remained less pronounced [12]. However, after completion of recruitment and inclusion of the full sample, the previously observed positive effect was no longer maintained.

Several factors may explain the absence of clear between-group differences. The sample size, although comparable with previous trials, may have been insufficient to detect small effects. All participants received intensive inpatient treatment, which itself produces substantial improvement and may reduce the relative contribution of additional interventions. The relatively short follow-up period may also have limited the ability to detect delayed neuromodulatory effects.

Concomitant antidepressant use may have also influenced the response to tDCS. In our study, the antidepressant and its dose remained unchanged for at least one month before enrollment and throughout the trial. This reduced the influence of recent treatment changes but did not rule out an interaction between antidepressants and tDCS. The sample was too small to examine outcomes according to medication use or to test this interaction. The possible contribution of antidepressant treatment to the observed effects therefore remains uncertain.

Interpretation of questionnaire-based outcomes should also be made with caution. A substantial part of the assessment relied on self-report measures, which may be influenced by current mood, level of insight into the illness, and willingness to disclose symptoms. In patients with AN, responses may be affected by denial, shame, fear of weight gain, or attempts to present themselves in a socially acceptable manner. These factors may reduce the sensitivity of questionnaire-based instruments to detect subtle treatment effects and may partly contribute to the lack of clear differences between study groups. An additional limitation is that the EAT-26 was developed primarily as a screening instrument rather than specifically as a measure of treatment-related change. Although its selection was justified by the constraints of the inpatient setting, the three-week intervention and two-week follow-up may have been too short to capture stable changes in self-reported eating disorder psychopathology. Future inpatient studies should consider longer follow-up and instruments specifically adapted for monitoring treatment-related change under structured hospital conditions.

The study has several strengths, including the randomized double-blind sham-controlled design, a relatively intensive stimulation protocol. Inclusion of both clinical and biological outcome measurements, and their integration in the analysis, is another major strength of the study. Conducting the study in a single inpatient center ensured consistency of treatment conditions and allowed careful monitoring of safety and adherence.

Despite these positive aspects, some limitations should be noted. Blinding success was not empirically assessed by asking participants or outcome assessors to guess treatment allocation at the end of the intervention. Therefore, although participants and assessors were intended to remain blinded, the effectiveness of blinding cannot be confirmed. The relatively small, single-center sample of female, predominantly adolescent inpatients and the short follow-up period limit the generalizability of the findings to males, adults, outpatients, other treatment settings, and longer-term clinical outcomes.

Another issue that should be highlighted is the effect of a small sample size and a homogeneous group on the obtained results in regression analysis, as limited data increases the risk of fitting spurious patterns. It should be noted that this was only a supplementary, rather than primary, analysis intended as a starting point or hypothesis for future studies by us and other researchers.

In conclusion, the results of this randomized study did not demonstrate that tDCS, when used as an adjunctive intervention in the treatment of AN, produced greater short-term symptom reduction than sham stimulation alongside standard treatment. The 2-week follow-up period does not allow conclusions regarding the maintenance of clinical improvement or relapse prevention. Findings concerning stress-related and metabolic processes require confirmation in larger and more diverse samples, across different treatment settings and with substantially longer follow-up periods. Future studies should also investigate whether patient characteristics or individualized stimulation parameters can identify subgroups that may benefit from adjunctive tDCS.

## Figures and Tables

**Table 2:** Distribution and daily doses of concomitant antidepressants by study group.

Drug Class	Medication	AG, *n*; Daily Dose	SC, *n*; Daily Dose
SSRI	Sertraline	75 mg/day, *n* = 1; 100 mg/day, *n* = 3; 150 mg/day, *n* = 8	50 mg/day, *n* = 1; 75 mg/day, *n* = 1; 100 mg/day, *n* = 3; 150 mg/day, *n* = 6; 200 mg/day, *n* = 2;
SNRI	Duloxetine	30 mg/day, *n* = 1	30 mg/day, *n* = 1; 60 mg/day, *n* = 1
TCA	Doxepine	10 mg/day, *n* = 1; 25 mg/day, *n* = 2; 50 mg/day, *n* = 1	10 mg/day, *n* = 1; 20 mg/day, *n* = 1; 25 mg/day, *n* = 4; 50 mg/day, *n* = 2
TCA	Clomipramine	125 mg/day, *n* = 1	—
TCA	Opipramol	—	50 mg/day, *n* =1
—	No antidepressant treatment	*n* = 7	*n* = 2

SSRI, selective serotonin reuptake inhibitor; SNRI, serotonin–norepinephrine reuptake inhibitor; TCA, tricyclic antidepressant. AG—active group, SG—sham group.

**Table 3:** Changes in the primary EAT-26 outcome and secondary psychological outcomes following tDCS.

Outcome	Group	V1 (Baseline)	V2 (Post-Intervention)	V3 (Follow-Up)	F (Group × Time); df	*p*; η^2^p	Post hoc Comparisons
EAT-26	AG	3.15 ± 21.86	20.20 ± 18.09	12.75 ± 11.03	0.806; 2	0.45; 0.02	V1 vs. V2 *p* = 0.023 V1 vs. V3 *p* < 0.001
	SG	28.19 ± 21.8	19.86 ± 20.01	15.43 ± 17.35			V1 vs. V2 *p* = 0.006
SES	AG	23.15 ± 6.8	24.5 ± 5.95	23.58 ± 5.06	1.9055; 2	0.156; 0.05	N/S
	SG	23.75 ± 5.85	23.95 ± 7.83	26 ± 5.66			N/S
BDI	AG	18.53 ± 13.3	17.8 ± 12.9	16.95 ± 14.01	1.7070; 2	0.189; 0.04	N/S
	SG	20 ± 12.78	15.29 ± 12.28	14.1 ± 10.09			V1 vs. V2 *p* = 0.005 V1 vs. V3 *p* = 0.002
PSS-10	AG	23.55 ± 9.24	20.8 ± 7.06	21.32 ± 6.69	0.24; 2	0.787; 0.01	N/S
	SG	23.43 ± 8.1	21.95 ± 7.59	21.89 ± 7.7			N/S

Data presented as mean and standard deviation; df—degree of freedom; η^2^p—partial eta-squared; EAT-26—Eating Attitudes Test; SES—Rosenberg Self-Esteem Scale; BDI—Beck Depression Inventory; PSS-10—Perceived Stress Scale; AG—active group; SG—sham group; N/S—not significant.

**Table 4:** Changes in exploratory biological outcomes following tDCS.

Variables	AG		SG	
	V1	V2	V1	V2
Cortisol (ng/mL)	31.06 (0.25–217.1); 43.40	30.72 (0.25–258.7); 35.94	35.53 (4.23–124.6); 22.67	35.53 (3.56–110.5); 15.52
NGF (pg/mL)	397.51 (290.9–1705.7); 166.92	383.89 (306.12–1979.2); 117.71	414.2 (295.26–1394.2); 220.48	411.08 (301.78–1192.3); 164.62
NT-3 (ng/mL)	4.84 (3.28–34.3); 3.29	4.41 (3.64–32); 1.72	4.99 (3.07–21.4); 2.30	4.78 (3.72–17.7); 3.14
NT-4 (ng/mL)	13 (9.69–119.4); 11.27	12.14 (9.48–112.8); 6.00	14.86 (10.11–70.3); 7.91	13.87 (10.11–49); 10.76
BDNF (ng/mL)	21.98 (12.61–36.8); 7.42	21 (6.22–38.8); 6.35	19.66 (10.28–28); 5.95	20.04 (17.48–27); 4.63
Visfatin (ng/L)	27.01 (20.71–146.8); 18.34	27.01 (21.11–175.3); 8.39	27.15 (20.71–110.2); 11.77	27.86 (20.71–87.4); 17.34
Leptin (ng/L)	3.54 (0.18–13.8); 6.63	9.78 (0.43–54.6); 11.45	3.09 (0.18–53.7); 8.57	14.78 (0.18–180.9); 29.88

Data presented as medians with minimum and maximum values and interquartile range; AG—active group, SG—sham group.

**Table 5:** Changes in the secondary anthropometric outcome (BMI) following tDCS.

Variable	AG			SG		
	V1	V2	V3	V1	V2	V3
BMI (kg/m^2^)	14.48 (13.22–17.25); 1.65	16.81 (13.26–18.03); 1.94	17.24 (14.14–18.54); 2.16	15.45 (13.43–17.35); 1.87	16.58 (14.18–18.75); 1.70	17.07 (15.39–19.61); 1.93

Data presented as medians with minimum and maximum values and interquartile range; AG—active group, SG—sham group.

## Data Availability

The data presented in this study are available on request from the corresponding author.

## Supplementary Materials

The following supporting information can be downloaded at https://www.mdpi.com/article/10.3390/jcm15176831/s1, File S1: CONSORT 2025 checklist of information to include when reporting a randomised trial.

## Abbreviations

The following abbreviations are used in this manuscript:

AN	Anorexia nervosa
tDCS	Transcranial direct current stimulation
ICD-11	International Classification of Diseases
DSM-5	Diagnostic and Statistical Manual of Mental Disorders
BMI	Body mass index
DLPFC	Dorsolateral prefrontal cortex
BDNF	Brain-derived neurotrophic factor
HPA	Hypothalamic–pituitary–adrenal
SSRIs	Selective serotonin reuptake inhibitors
SNRIs	Serotonin–norepinephrine reuptake inhibitors
TCAs	Tricyclic antidepressants
V0	Screening visit
V1	Baseline visit
V2	Post-intervention visit
V3	Follow-up visit
EAT-26	Eating Attitudes Test
BDI	Beck Depression Inventory
PSS-10	Perceived Stress Scale
SES	Rosenberg Self-Esteem Scale
